# Acute Pancreatitis (AP) in Pregnancy and Its Complications From an Anesthesia Perspective: A Case Report

**DOI:** 10.7759/cureus.38913

**Published:** 2023-05-11

**Authors:** Ahmed K Alanzi, Amir Fouad, Samar Ghazzal, Shahid Adeel, Ahmed Eldesokey

**Affiliations:** 1 Anesthesia and Critical Care, King Hamad University Hospital, Muharraq, BHR

**Keywords:** pregnancy, acute pancreatitis, anesthesia, lipidemia, pancreas, abdominal pain

## Abstract

Acute pancreatitis (AP) is a rare event in pregnancy that is characterized by a sudden and severe inflammation of the pancreas. The clinical manifestation of AP during pregnancy is highly variable ranging from a mild form to a severe and potentially life-threatening presentation. We share a case of a 29-year-old female (gravida II, para I) who presented in her 33rd gestational week. The patient complained of upper abdominal pain and nausea. Her previous history revealed that she had four episodes of vomiting (food-containing, non-projectile) at home. Her uterine tone was normal, and her cervix was closed. Her white blood cell count was 13,000/mm^3^, and her C-reactive protein (CRP) level was 65 mg/L. She underwent an emergency laparotomy due to suspected acute appendicitis; however, no peritonitis was found intraoperatively. Further blood tests showed high levels of triglyceride at 87.5 mmol/L. The electrophoretic pattern of lipoprotein was consistent with type V hyperlipoproteinemia. An abdominal computed tomography (CT) confirmed the diagnosis of acute pancreatitis. During follow-up after one month, the patient showed triglyceride levels at 4.75 mmol/L and cholesterol at 6.07 mmol/L. Acute pancreatitis due to hypertriglyceridemia is a rare finding; nonetheless, it should be considered as a potential etiology in pregnant patients with nonobstructive abdominal pain.

## Introduction

Acute pancreatitis (AP) is a rare yet serious medical condition in pregnancy that affects approximately one in 1,000 to one in 5,000 pregnancies [1,2]. This disorder is characterized by acute inflammation of the pancreas, subsequently leading to the activation of digestive enzymes that damage surrounding tissues and organs [3]. The incidence of acute pancreatitis is higher in the later stages of pregnancy, with most cases reported in the second and third trimesters [4,5]. The manifestation of acute pancreatitis in pregnancy is highly variable and ranges from mild to severe, involving necrotic processes, pseudocyst formation, abscess formation, and systemic dysfunction [6]. The evaluation of the severity of acute pancreatitis in pregnancy is complicated by hematological and biochemical changes that are intrinsic to pregnancy. This makes the management of this condition a challenging task, as it involves not only the well-being of the mother but also the welfare of the fetus.

A systematic review of 8,466 pregnant patients reported maternal mortality ranging from 0 to 12.12/100 pregnancies, fetal loss from 0% to 23.08%, and neonatal mortality from 0 to 75.5/1000 neonatal live births [7]. The primary cause of AP in pregnancy is of biliary origin (gallstones), accounting for 70% of cases, whereas significant contributors include long-standing alcohol use and hypertriglyceridemia [8]. Pregnancy does not increase the risk of pancreatitis. However, several physiological modifications during pregnancy can increase the risk of gallstone formation and biliary sludge accumulation [8]. These alterations are attributed to various factors, such as increased bile acid pool size, diminished enterohepatic circulation, altered bile acid composition, and reduced gallbladder contractility [9]. The steroid hormones of pregnancy, such as progesterone and estrogens, exacerbate the risk of biliary-induced acute pancreatitis by contributing to an altered bile acid composition and decreased gallbladder motility [10]. Other contributing factors include hyperlipidemia, alcohol abuse, hyperparathyroidism, connective tissue diseases, abdominal surgery, abdominal injuries, and iatrogenic factors such as medication use [11]. Hypertriglyceridemia is the third leading cause of acute pancreatitis in pregnancy, affecting approximately 4% of all cases [12]. The increased plasma lipid levels observed during pregnancy are a physiological response to hormonal changes, although they are insufficient to cause acute pancreatitis [13]. The underlying mechanism of biliary-induced acute pancreatitis during pregnancy includes duct obstruction, which leads to pancreatic hyperstimulation and increased pressure, trypsin reflux, and activation of trypsin within pancreatic acinar cells. This results in autodigestion of the pancreas and local inflammation [14]. We present the case of a previously healthy pregnant 29-year-old female who was diagnosed with AP.

## Case presentation

A 29-year-old female (gravida II, para I) presented to the emergency department in the 33rd gestational week complaining of upper abdominal pain and nausea, which were not associated with vaginal leakage or bleeding. Prior to the presentation, she had a cesarean section in India (reason unknown). Her previous history revealed that she had four episodes of vomiting (food-containing, non-projectile) at home associated with abdominal pain. Upon admission, the patient’s vital signs were stable, and she did not show any signs of fever. She experienced fetal movements, and a gynecological examination revealed no evidence of premature labor. The examination revealed the presence of a single viable fetus with a cephalic presentation and an anterior placenta. The amniotic fluid volume was within normal limits, with an estimated fetal weight of 2.2 kg. Her uterine tone was normal, and her cervix was closed. However, her white blood cell count was 13,000/mm^3 ^(reference range: 4,000-9,000/mm^3^), C-reactive protein (CRP) level was 65 mg/L (reference range: 0-5 mg/L), amylase level was 858 U/L, and lipase level was 8,650 U/L (Table 1).

**Table 1:** Different parameters of the patient at the initial assessment.

Measurement	Result	Reference range
White blood cell count	13,000/mm^3^	4,000-9,000/mm^3^
C-reactive protein	418 mg/L	0-5 mg/L
Amylase	858 U/L	30-100 U/L
Lipase	8,650 U/L	8-78 IU/L
HbA1c	11.6%	4%-5.6%
Triglycerides	87.5 mmol/L	0-1.8 mmol/L
Procalcitonin	1.19 μg/L	0-0.05 μg/L
Lactate	5.78 mmol/L	0.63-2.44 mmol/L
Lipase activity	1,176 IU/L	8-78 IU/L
Neutrophils	92%	50%-72%
Hematocrit	36.2%	36%-42%

Immediate medical consultation was performed, and the patient was admitted and kept nil per oral, and intravenous hydration was commenced. Although the patient initially had no history of diabetes, her HbA1c level was 11.6%, which was assessed as poorly controlled diabetes mellitus. She was continuously given an insulin infusion of 0.2 units/kg/hour. The day after admission, the patient developed sudden tachypnea and tachycardia (130-140 bpm). ECG showed T-wave inversion in lead 3 and S-waves in lead 1, and lower leg Doppler US was performed, which was negative for deep venous thrombosis (DVT). Computed tomography pulmonary angiography (CTPA) and ventilation/perfusion (V/Q) scans were also recommended. The patient was thoroughly informed of the advantages and disadvantages of CTPA and V/Q scans. When she was explained that the V/Q scan was safer for her fetus, she opted for a V/Q scan; however, it turned out negative. At this stage, the patient was transferred to the ICU for closer monitoring. Furthermore, the patient developed signs of peritonitis. Abdominal ultrasound showed chylous ascites, which was drained immediately. Blood tests showed high levels of triglyceride at 87.5 mmol/L, CRP at 418 mg/L, procalcitonin at 1.19 μg/L, lactate at 5.78 mmol/L, lipase activity at 1,176 IU/L, and white blood cells at 13,470/mm^3^, with 92% being neutrophils. Her hematocrit level was 36.2% (Table 1).

The electrophoretic pattern of lipoproteins was consistent with that of type V hyperlipoproteinemia. Drainage fluid showed high amylase (3,520 IU/L) and lipase (39,160 IU/L) levels. Owing to impending signs of fetal hypoxia on cardiotocography, an emergency cesarean section was performed. A full assessment was performed by the anesthesiologist in the labor room, and a dextrose 10% insulin + potassium chloride (KCL) infusion was performed. The patient had tachycardia at a rate of 134/minute, was dyspneic on a face mask in a semi-sitting position, and had oxygen saturation between 95% and 97%. The patient was immediately transferred to the operating room. All American Society of Anesthesiologists (ASA) monitors were attached, and baseline hemodynamics were obtained. Furthermore, a 20-G cannula was secured to the left dorsum of the hand. The previous IV fluid was switched to Ringer’s lactate and infused through a warmer fluid at a rate of 120 mL/hour. The patient underwent general anesthesia with rapid sequence induction, which involved using the Sellick maneuver to help secure the airway. She was administered propofol 150 mg (2 mg/kg) and succinylcholine 100 mg (1.5 mg/kg). Ketamine 20 mg was given after the induction along with atracurium 50 mg (0.7 mg/kg) after the wear off of the succinylcholine effect. After the muscle spasms were stopped, an endotracheal tube with a diameter of 7 mm and a length of 19 cm was inserted through the mouth to secure the airway. Intubation was confirmed by direct visualization and positive capnography, and bilateral auscultation of the chest was equal and clear. The anesthesia was maintained with volume-controlled mode targeted tidal volume of 6-8 mL/kg, respiratory rate of 13 breaths/minute, positive end-expiratory pressure (PEEP) of 5 mmHg, and inhalational sevoflurane at a minimum alveolar concentration (MAC) of 0.8-1. An arterial line was inserted in the left radial artery, and a triple-lumen 7-French central line was inserted in the right internal jugular vein using ultrasound guidance under an aseptic technique.

**Figure 1:**
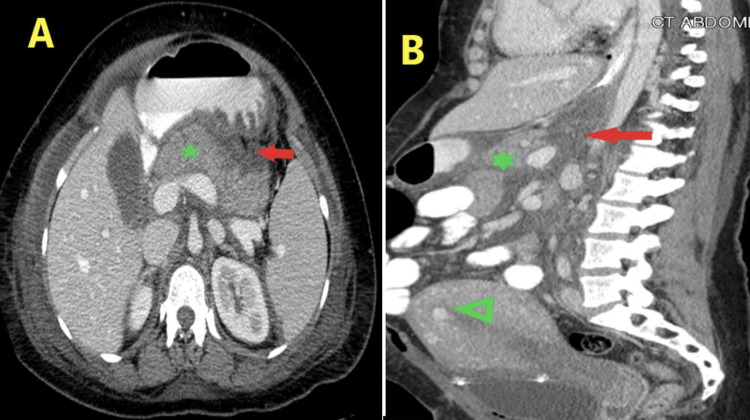
CT of the abdomen with contrast. A: Diffusely edematous and swollen pancreas (green star) with loss of its feathery appearance and associated smudged surrounding fat planes and mild prominence of the CBD. B: Partially encysted peripancreatic and lesser sac fluid collection (red arrow) and postdelivery bulky uterus (green arrowhead). CT: computed tomography, CBD: common bile duct

**Figure 2:**
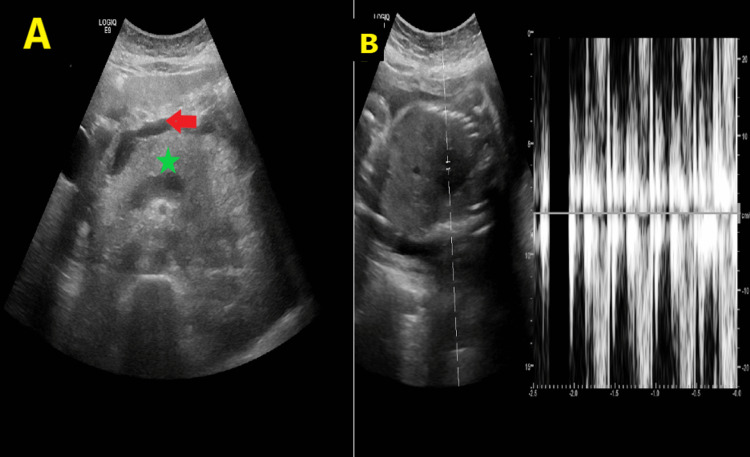
A: Abdominal ultrasound study for the pregnant patient at 33 weeks showing diffusely edematous and swollen pancreas (green star) with loss of its feathery appearance and associated peripancreatic free fluid collection (red arrow), impressive of acute pancreatitis. B: Gravid uterus showing single viable intrauterine fetal pregnancy.

After the delivery of the fetus, oxytocin (10 units) was administered as a bolus, and an infusion of 40 units in 1 L of Ringer’s lactate was administered at a rate of 125 mL/hour. Carbetocin 100 µg was needed as well. Prophylactic cefazolin 2 g and metronidazole 500 mg were given. Dexamethasone 8 mg and ondansetron 4 mg were received as well. Due to her low hemoglobin, which was 9.1 before fluid shifting and was 7.9 because of bleeding intraoperatively, she received one unit of packed red blood cells and 2 L of Ringer’s lactate postoperatively. The blood pressure was maintained intraoperatively with intravenous fluids, and the mean arterial pressure (MAP) was above 60 mmHg. Postoperatively, she was sent to the intensive care unit for observation, intubated, ventilated, and sedated with remifentanil infusion at 0.3 µg/kg. Furthermore, chest radiography was performed to confirm the placement of the central line. She delivered a healthy newborn female and was admitted to the neonatal intensive care unit (NICU) for two weeks and discharged in good condition with an uneventful stay. Abdominal computed tomography (CT) confirmed the diagnosis of acute pancreatitis along with turbid intraperitoneal collection (Figure 1 and Figure 2). Despite conservative treatment with heparin and insulin infusion, triglyceride levels remained high. Three sessions of plasmapheresis were performed to reduce hypertriglyceridemia. The patient was eventually discharged after 30 days of hospitalization with a triglyceride level of 4.75 mmol/L and cholesterol level of 6.07 mmol/L (reference range: 0-5.2 mmol/L).

## Discussion

We report a case of hypertriglyceridemia-induced AP in a pregnant patient in her third trimester, with no prior history of any lipid metabolism disorder. Our findings suggest that pregnancy is an initiating factor of hypertriglyceridemia. Gallstones and hypertriglyceridemia are the two major factors implicated in the development of AP [15]. The correlation between hypertriglyceridemia and acute pancreatitis has been firmly established in the medical literature [16]. Despite this, mild-to-moderate elevations in triglycerides are frequently observed in almost 50% of AP cases and are generally considered to be a secondary phenomenon rather than a causative factor [17]. The current scientific consensus suggests that serum triglyceride concentrations > 11.3 mmol/L are necessary for the development of acute pancreatitis [18]. A prospective study of 716 participants showed that serum triglycerides of 11.3 mmol/L are significantly associated with the possibility of AP [19]. The peak concentration of triglycerides during pregnancy is observed in the third trimester and may exhibit an increase of up to 2-4 times the normal levels. Nevertheless, the levels rarely surpassed 11 mmol/L. A similar pattern is typically observed in the elevation of cholesterol concentration [20].

The diagnosis of AP associated with pregnancy presents numerous challenges and controversies in the literature [21]. Our patient manifested with symptoms of upper abdominal pain and nausea. Similar findings were reported by Luo et al. [22], who investigated 121 cases of AP, and the most reported symptom was upper abdominal pain. Pregnant patients often present with symptoms commonly observed in AP, such as nausea, vomiting, abdominal discomfort, or pain. However, the clinical evaluation of the acute abdomen during pregnancy can be complicated because of the anatomical displacement of abdominal organs by the gravid uterus [23]. This displacement leads to the reduced manifestation of classic signs and symptoms of peritonitis due to the lifting of the abdomen from the area of inflammation. Additionally, the presence of the uterus impairs the movement of the omentum to the site of inflammation, which makes the clinical picture ambiguous. This can result in misdiagnosis or unnecessary non-obstetric surgical interventions, which are associated with an increased risk of premature labor [24]. In contrast to our case, Abdullah et al. [25] reported a case of AP during the first trimester that was initially diagnosed as a ruptured ectopic pregnancy and corpus luteal cyst. The utilization of imaging techniques is crucial in the identification of acute pancreatitis, as it facilitates the determination of the underlying cause and classification of the severity of the condition. In this specific case, the diagnosis of acute pancreatitis in the subject was primarily established through the evaluation of clinical symptoms, laboratory indicators of pancreatic inflammation, and the results of ultrasound imaging.

Anesthesia management in pregnant AP patients requires special considerations due to the unique physiologic changes that occur during pregnancy and the potential complications associated with AP. Several studies have supported the use of epidural anesthesia in pregnant patients undergoing C-section, as it provides excellent analgesia, minimizes the need for opioids, and allows for early mobilization [26]. Dogra et al. [27] investigated the efficacy of epidural anesthesia compared to general anesthesia on maternal-fetal outcomes and pain relief. Their findings showed that epidural anesthesia was better compared to intravenous anesthesia in terms of pain management and recovery of patients. Similar findings were shared by Khan et al. [28] in support of the efficacy of epidural anesthesia; however, they also reported some fetal complications, including prematurity, respiratory distress, and babies requiring noninvasive ventilation in patients treated with tramadol. However, Pandey et al. [29] propagated that general anesthesia could provide better hemodynamic stability in the presence of sepsis, during the use of anticoagulants such as heparin or epoprostenol for continuous venovenous hemofiltration (CVVH), and when there is a potential need for controlled postoperative ventilation. Furthermore, epidural anesthesia in patients with AP requires caution due to the potential for epidural abscess or hematoma formation, particularly in the presence of coagulopathy or thrombocytopenia [30]. In this case, we successfully delivered the baby via cesarean section under general anesthesia while closely monitoring the patient’s condition.

## Conclusions

In conclusion, our case highlights the importance of considering hypertriglyceridemia as a possible cause of acute pancreatitis in pregnant women, particularly in the third trimester when serum triglyceride concentrations may be elevated. The diagnosis of AP associated with pregnancy is challenging because of the anatomical displacement of the abdominal organs and the altered presentation of symptoms. The utilization of imaging techniques is crucial for identifying and evaluating these conditions. Our findings suggest that pregnancy may be an initiating factor for hypertriglyceridemia and that triglyceride levels should be closely monitored during pregnancy to prevent the development of AP. Additionally, early recognition and proper management of AP during pregnancy are crucial to avoid potential consequences such as misdiagnosis and unnecessary non-obstetric surgical interventions.
